# Marked hydronephrosis and hydroureter after distigmine therapy in an adult male patient with paraplegia due to spinal cord injury: a case report

**DOI:** 10.4076/1757-1626-2-7333

**Published:** 2009-08-06

**Authors:** Subramanian Vaidyanathan, Paul Mansour, Bakul M Soni, Peter L Hughes, Gurpreet Singh, Tun Oo

**Affiliations:** 1 Regional Spinal Injuries Centre, District General HospitalSouthport PR8 6PNUK; 2 Department of Cellular Pathology, District General HospitalSouthport PR8 6PNUK; 3 Department of Radiology, District General HospitalSouthport PR8 6PNUK; 4 Department of Urology, District General HospitalSouthport PR8 6PNUK

## Abstract

**Introduction:**

Distigmine, a long-acting anti-cholinesterase, is associated with side effects such as Parkinsonism, cholinergic crisis, and rhabdomyolysis. We report a spinal cord injury patient, who developed marked hydronephrosis and hydroureter after distigmine therapy, which led to a series of complications over subsequent years.

**Case presentation:**

A 38-year-old male developed T-9 paraplegia in 1989. Intravenous urography, performed in 1989, showed normal kidneys, ureters and bladder. He was prescribed distigmine bromide orally and was allowed to pass urine spontaneously. In 1992, intravenous urography showed bilateral marked hydronephrosis and hydroureter. Distigmine was discontinued. He continued to pass urine spontaneously.

In 2006, intravenous urography showed moderate dilatation of both pelvicalyceal systems and ureters down to the level of urinary bladder. This patient was performing self-catheterisation only once a day. He was advised to do catheterisations at least three times a day. In December 2008, this patient developed haematuriawhich lasted for nearly four months.. He received trimethoprim, then cephalexin, followed by Macrodantin, amoxicillin and ciprofloxacin. In February 2009, intravenous urography showed calculus at the lower pole of left kidney. Both kidneys were moderately hydronephrotic. Ureters were dilated down to the bladder. Dilute contrast was seen in the bladder due to residual urine. This patient was advised to perform six catheterisations a day, and take propiverine hydrochloride 15 mg, three times a day. Microbiology of urine showed *Klebsiella oxytoca*, *Pseudomonas aeruginosa*, and *Enterococcus faecalis*. Cystoscopy revealed papillary lesions in bladder neck and trigone. Transurethral resection was performed. Histology showed marked chronic cystitis including follicular cystitis and papillary/polypoid cystitis. There was no evidence of malignancy.

**Conclusion:**

Distigmine therapy resulted in marked bilateral hydronephrosis and hydroureter. Persistence of hydronephrosis after omitting distigmine, and presence of residual urine in bladder over many years probably predisposed to formation of polypoid cystitis and follicular cystitis, and contributed to prolonged haematuria, which occurred after an episode of urine infection. This case illustrates the dangers of prescribing distigmine to promote spontaneous voiding in spinal cord injury patients. Instead of using distigmine, spinal cord injury patients should be advised to consider intermittent catheterisation together with oxybutynin or propiverine to achieve complete, low-pressure emptying of urinary bladder.

## Introduction

In anaesthetised guinea pigs, distigmine increased intravesical pressure at the maximum flow rate and also decreased bladder compliance. Distigmine produced deterioration of voiding function by inducing contraction of the external urethral sphincter muscle and thereby increasing urethral resistance. Distigmine also caused deterioration of storage function of urinary bladder as well [1]. In clinical studies, distigmine has been shown to increase the tone of urethral sphincter [2]. We report a spinal cord injury patient who developed marked hydronephrosis and hydroureter after he was prescribed distigmine and allowed to void spontaneously. Persistence of hydronephrosis after omitting distigmine and presence of residual urine in bladder over many years probably predisposed to formation of polypoid cystitis and follicular cystitis, and contributed to prolonged haematuria, which occurred after an episode of urine infection. This case illustrates dangers of prescribing distigmine to promote spontaneous voiding in spinal cord injury patients.

## Case presentation

**Figure 1.**
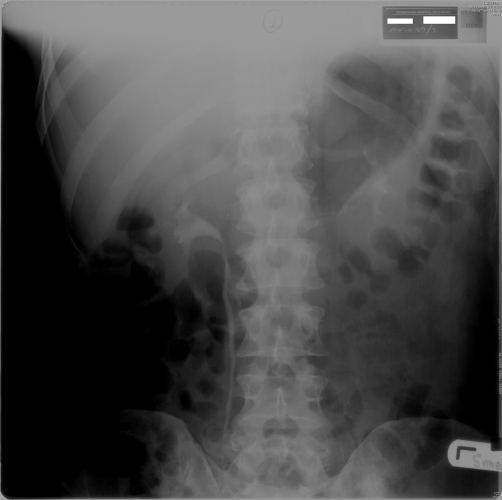
Intravenous urography - five minutes film (15 November 1989) showed prompt excretion of contrast and undilated pelvicalyceal systems.

**Figure 2.**
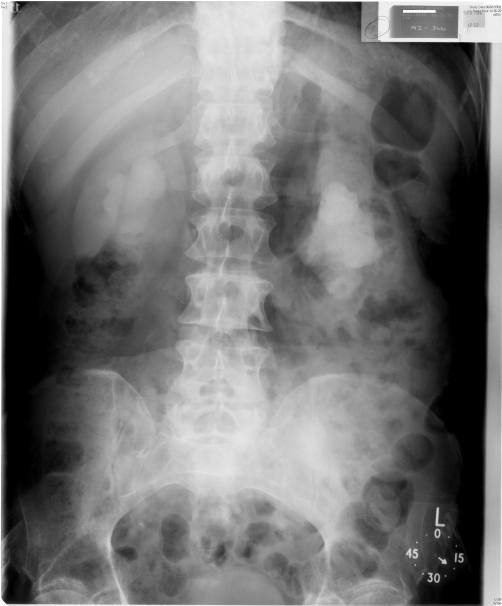
Intravenous urography (06 January 1992): Twenty minutes film showed marked bilateral hydronephrosis.

**Figure 3.**
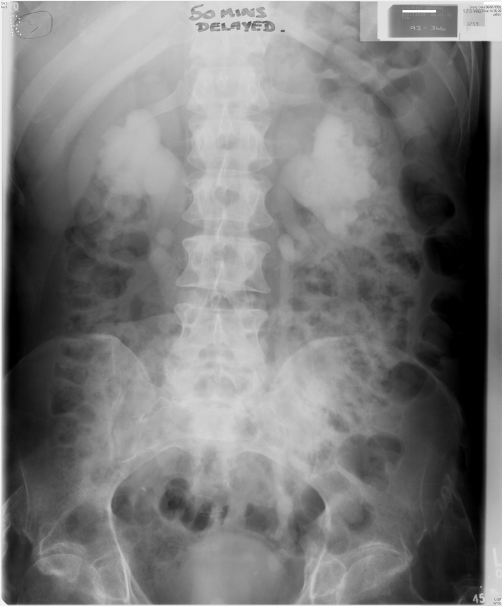
Intravenous urography (06 January 1992): Fifty minutes film showed marked bilateral hydronephrosis and hydroureter.

**Figure 4.**
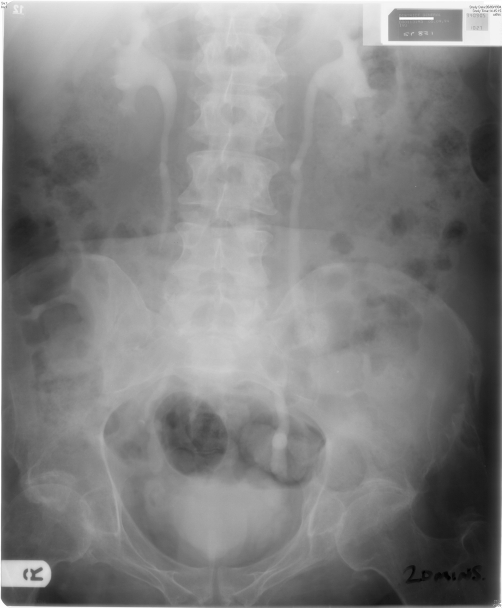
Intravenous urography (05 September 1994): Twenty minutes film showed mild dilatation of renal pelvis and both ureters.

A previously fit, 38-year-old, white, British male fell from a tree in 1989 whilst fixing a tree-swing for the children. He sustained fracture of T-10 vertebra, fracture of distal end of left clavicle and paraplegia below T-9. He was treated conservatively. Intravenous urography, performed on 15 November 1989, showed normal kidneys, ureters and bladder (Figure 1). He was prescribed distigmine bromide orally and was allowed to pass urine spontaneously. In January 1992, intravenous urography showed bilateral hydronephrosis and hydroureter (Figure 2 and Figure 3). The bladder outline appeared normal. In February 1992, ultrasound examination revealed bilateral hydronephrosis; good renal cortical thickness, urinary bladder was thick-walled. Cystogram was performed on 02 September 1992. The bladder was thick walled and trabeculated with diverticula. Intravenous urography, performed on 05 September 1994, showed no radio opaque calculi. There was fullness of pelvicalyceal systems. Ureters were mildly distended throughout their length (Figure 4). Diverticula were arising from urinary bladder, which was only faintly delineated by contrast. This patient was advised to perform self-catheterisations 4-5 times a day.

**Figure 5.**
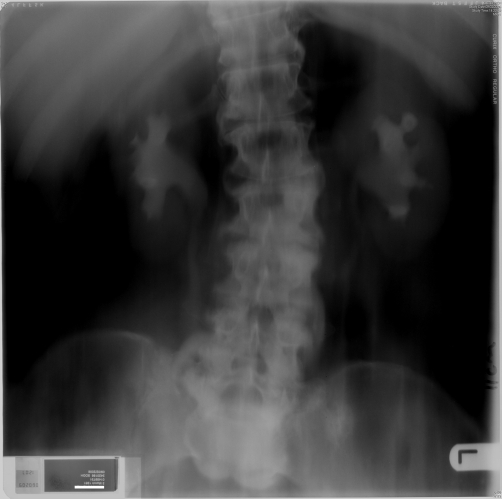
Intravenous urography (09 February 2006): Tomogram showed dilated bilateral renal pelves with clubbing of calyces and calculus in lower pole of left kidney.

**Figure 6.**
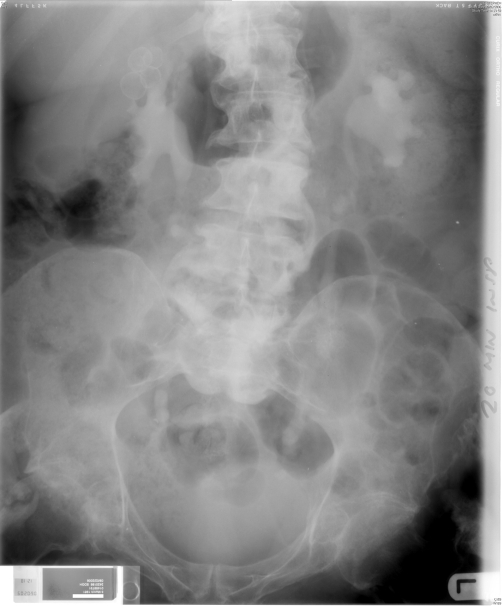
Intravenous urography (09 February 2006): Twenty minutes film showed bilateral hydronephrosis, mild hydroureter and calculus in lower pole of left kidney.

Intravenous urography, performed on 09 February 2006, showed 12 mm diameter calculus in lower pole of left kidney. There was moderate dilatation of both pelvicalyceal systems and ureters down to the level of urinary bladder (Figure 5 and Figure 6). Radio opaque gallstones were present. This patient was performing self-catheterisation once a day. He was advised to consider whether he could do catheterisations at least three times a day. His blood pressure was 171/100 mm Hg. He was prescribed Doxazosin modified-release 4 mg once daily. Doxazosin, an alpha-adrenergic blocker, would help to lower blood pressure and facilitate emptying of urinary bladder.

This patient started passing blood in urine on 05 December 2008. He went to a drop-in centre where he was prescribed trimethoprim. Trimethoprim did not have any effect. On 12 December 2008, he was prescribed Cephalexin. On 19 December 2008, this patient was prescribed Macrodantin 100 mg, one, twice a day. He developed nausea after taking Macrodantin. On 23 December 2008, he was prescribed amoxicillin. On 31 December 2008, this patient was prescribed ciprofloxacin 500 mg, one twice a day for five days. He was referred to local hospital where flexible cystoscopy was performed. Cystoscopy showed markedly distended, trabeculated bladder. Ultrasound revealed bilateral hydronephrosis, more so on left side. There was some irregularity on the left wall of urinary bladder but flexible cystoscopy revealed only marked trabeculations. He was advised to perform intermittent catheterisations at least two to three times a day.

**Figure 7.**
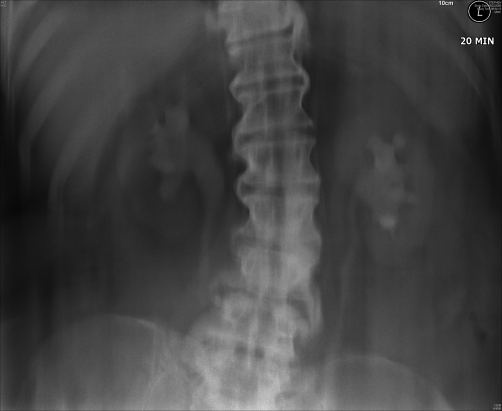
Intravenous urography (18 February 2009): Twenty minutes film 10 cm tomogram showed bilateral hydronephrosis and calculus in lower pole of left kidney.

**Figure 8.**
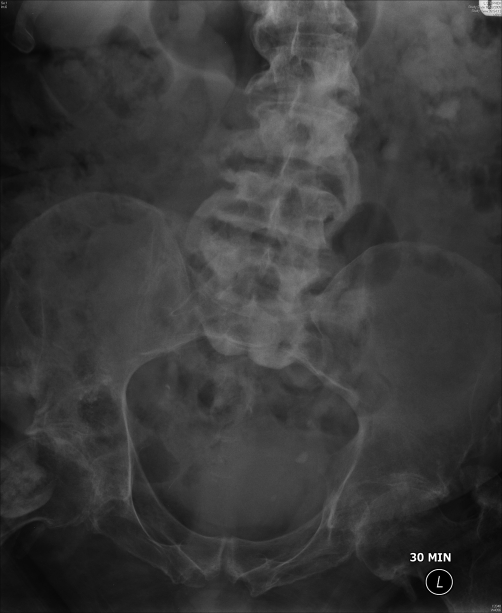
Intravenous urography (18 February 2009): Thirty minutes film showed mild hydronephrosis and hydroureter. There was dilute contrast in urinary bladder.

Intravenous urography, performed on 18 February 2009, showed calculus at the lower pole of left kidney. Radio opaque gallstones were present. Both kidneys were moderately hydronephrotic. Ureters were dilated down to the bladder. Dilute contrast was seen in the bladder due to residual urine (Figure 7 and Figure 8). Bladder outline could not be assessed.

This patient was advised to perform six catheterisations a day. He was prescribed propiverine hydrochloride 15 mg, three times a day.

**Figure 9.**
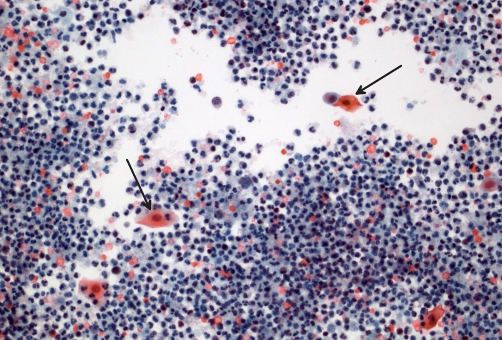
Urine cytology (Papanicolau stain) showing heavy background of polymorphs and red cells, with scattered benign epithelial cells still identifiable (arrows).

Cytospin preparations of urine, sent on 18 February 2009, showed very large numbers of polymorph leucocytes, suggesting an acute urinary tract infection. Red blood cells, benign urothelial cells and squamous cells were identified. No malignant cells were present (Figure 9).

Microbiology of urine (05 March 2009) showed growth of *Klebsiella oxytoca*, *Pseudomonas aeruginosa*, and *Enterococcus faecalis*.

**Figure 10.**
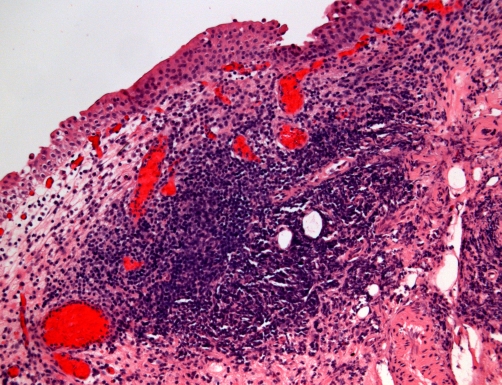
Histology of bladder mucosa (H&E stain) showing marked chronic inflammation including a poorly-defined lymphoid follicle centrally, constituting follicular cystitis. Benign surface urothelium is present at top.

**Figure 11.**
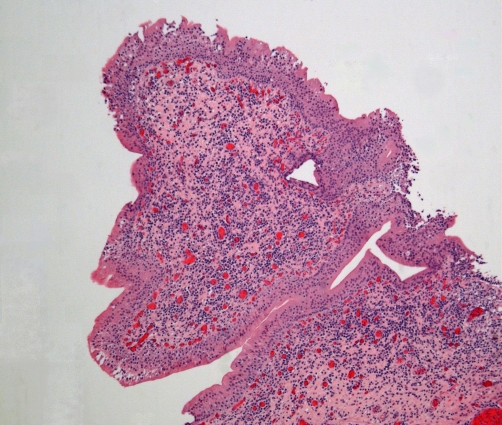
Low-power histology view of a broad, polypoid projection of mucosa covered by benign urothelium and showing marked inflammation of its stroma, constituting polypoid cystitis.

Cytospin preparations of urine, sent on 25 March 2009, showed benign urothelial cells, red blood cells, and large numbers of neutrophils suggestive of acute infection. No malignant cells were seen. Cystoscopy, performed on 27 March 2009, showed extensive papillary lesions in bladder neck and trigone extending to both lateral walls; transurethral resection was carried out. Histology of biopsies taken from bladder neck, trigone and lateral walls showed bladder mucosa, submucosa and muscularis propria. Features of marked chronic cystitis including follicular cystitis (Figure 10) and papillary/polypoid cystitis (Figure 11) were present. There was no significant acute inflammation. There was no evidence of carcinoma in situ, papillary or invasive carcinoma. A sample of urine taken on 06 May 2009 showed growth of coliform and Pseudomonas aeruginosa, both sensitive to ciprofloxacin and gentamicin.

## Discussion

Distigmine inhibits the breakdown of acetylcholine. Bethanechol is a parasympathomimetic drug. Yalla and associates [3] studied 70 spinal cord injury male patients and concluded that bethanechol chloride administration might exaggerate detrusor sphincter dyssynergia and therefore, injudicious use of bethanechol could be detrimental to the urinary tract. Yamanishi and associates [4] investigated one hundred and nineteen patients with under-active bladder, who were assigned to three groups: the cholinergic group, consisting of 40 patients taking bethanechol chloride (60 mg/day) or distigmine bromide (15 mg/day); the alpha-blocker group, consisting of 38 patients taking urapidil (60 mg/day); and the combination group, consisting of 41 patients taking both a cholinergic drug and an alpha-blocker. The effectiveness of each therapy was assessed 4 weeks after initialization of the therapy. The average and maximum flow rates did not increase significantly after monotherapy with either the cholinergic drug or the alpha-blocker, but they significantly increased after combination therapy compared to baseline values (P = 0.0033 and P= 0.0004, respectively). Post-void residual volume did not decrease significantly after the cholinergic drug therapy, but decreased significantly after the alpha-blocker (P = 0.0043) and the combination therapies (P = 0.0008). Yamanishi and associates [4] concluded that combination therapy with a cholinergic drug and an alpha-blocker appeared to be more useful than monotherapy for the treatment of underactive detrusor.

These two studies show clearly that use of cholinergic drug, bethanechol or cholinesterase inhibitor*,* distigmine bromide may prove detrimental to urinary tract. Such deterioration of upper urinary tract indeed happened in our patient. After taking distigmine bromide and allowing the bladder to drain into a penile sheath, our patient developed massive bilateral hydronephrosis. In hindsight, we realise our folly of prescribing distigmine as monotherapy. Either we should have recommended dual therapy consisting of distigmine and an alpha-adrenoceptor blocking drug or, preferably, we should have advised intermittent catheterisations along with anticholinergic agent.

Side effects of distigmine such as Parkinsonism [5], cholinergic crisis [6], and rhabdomyolysis [7], are documented in literature. We report a serious urological side effect of distigmine in a spinal cord injury patient. Not only this patient developed marked hydronephrosis and hydroureter after distigmine therapy, but also experienced a series of complications such as cystitis follicularis, polypoid cystitis, and haematuria over subsequent years.

Cystitis follicularis (follicular cystitis) is characterized by formation of lymphoid follicles in the lamina propria of the trigonal region of the bladder, and is considered to be the result of repeated bouts of urinary tract infection, usually bacterial, with other pathologic processes contributing to the development and prolongation of the infection. Cytologically it differs from chronic cystitis with prominent lymphocytosis by the presence of cellular elements from the germinal centres of lymphoid follicles, reminiscent of the cytologic findings in follicular cervicitis, with possible additional epithelial cytologic atypias from the overlying urothelium, which frequently undergoes reactive changes (hyperplastic, metaplastic, and ulcerative) [8]. In this patient, persistence of hydronephrosis, hydroureter and residual urine in urinary bladder predisposed to chronic urine infection with acute exacerbations, which resulted in formation of follicular cystitis.

Polypoid cystitis and its more chronic phase papillary cystitis, which results as a reaction to injury to the bladder mucosa, is a benign lesion mimicking various papillary urothelial neoplasms [9]. It has been hypothesised that in spinal cord injury patients, polypoid cystitis is associated with long-term indwelling catheters [10]. This case illustrates that in persons with spinal cord injury, who do not have long-term indwelling catheters, are still at risk for developing complications such as marked chronic cystitis and polypoid cystitis if they continue to retain large amounts of urine in bladder for a long duration.

In the presence of papillary/polypoid cystitis, follicular cystitis and marked chronic cystitis, this patient developed prolonged haematuria, which was precipitated by an episode of urine infection. All these complications could have been prevented had we established a regimen of complete, low-pressure emptying of urinary bladder by regular intermittent catheterisations along with oral oxybutynin or propiverine hydrochloride.

## Conclusion

Distigmine therapy resulted in marked bilateral hydronephrosis and hydroureter in this spinal cord injury patient with T-9 paraplegia. Persistence of hydronephrosis after omitting distigmine, and presence of residual urine in bladder over many years probably predisposed to formation of polypoid cystitis and follicular cystitis, and contributed to prolonged haematuria, which occurred after an episode of urine infection. This case illustrates the dangers of prescribing distigmine to promote spontaneous voiding in spinal cord injury patients. Instead of using distigmine, spinal cord injury patients should be advised to consider intermittent catheterisation together with oxybutynin or propiverine to achieve complete, low-pressure emptying of urinary bladder.
